# An Efficient Evaluation of F-doped Polyanion Cathode Materials with Long Cycle Life for Na-Ion Batteries Applications

**DOI:** 10.1038/s41598-017-13718-0

**Published:** 2017-11-01

**Authors:** Rasu Muruganantham, Yi-Tang Chiu, Chun-Chuen Yang, Chin-Wei Wang, Wei-Ren Liu

**Affiliations:** 1 Department of Chemical Engineering, Chung Yuan Christian University, Taoyuan City, Chungli, 32023 Taiwan ROC; 2 Department of Physics, Chung Yuan Christian University, Taoyuan City, Chungli, 32023 Taiwan ROC; 3 Neutron Group, National Synchrotron Radiation Research Center, Hsinchu City, 30076 Taiwan ROC

## Abstract

A series of Na_3−*x*_V_2_(PO_4−*x*_F_*x*_)_3_ (*x* = 0, 0.1, 0.15 and 0.3) polyanion cathode materials are synthesized via a sol-gel method. The optimal doping concentration of F in Na_3_V_2_(PO_4_)_3_ is 0.15 mol %. By neutron powder diffraction data, the chemical composition of as-synthesized material is Na_2.85_V_2_(PO_3.95_F_0.05_)_3_. The half-cell of Na_2.85_V_2_(PO_3.95_F_0.05_)_3_ cathode exhibits a stable discharge capacity of 103 mAh g^−1^ and 93% of capacity retention over 250 cycles without decay at 0.1 A g^−1^, which is higher than that of bare Na_3_V_2_(PO_4_)_3_ (98 mAh g^−1^). The high rate capability of Na_2.85_V_2_(PO_3.95_F_0.05_)_3_ is also dramatically enhanced via increase the conductivity of host material by F-doping. Moreover, the symmetrical Na-ion full-cell is fabricated using Na_2.85_V_2_(PO_3.95_F_0.05_)_3_ as cathode and anode materials. It is achieved that the good reversibility and superior cycling stability about 98% of capacity retention with ~100% of coulombic efficiency at 1.0 A g^−1^ throughout 1000 cycles. These results demonstrate that the optimal amount of Na_2.85_V_2_(PO_3.95_F_0.05_)_3_ is a distinctive potential candidate for excellent long-term cyclic stability with high rate low-cost energy storage applications.

## Introduction

Over the past decades onwards, Li-ion batteries have been widely used for various energy sectors such as portable electronics and transport applications. Conversely, the limited stock of lithium source availability and increasing cost may restrict their large-scale electrical energy storage systems. Numerous researchers have been focused on alternative batteries, such as Na-ion, K-ion, Al-ion, Mg-ion batteries and so on^1–4^. Among those batteries, sodium-ion batteries (SIBs) have been attracted significant interest as a promising alternative to lithium-ion batteries (LIBs) for merits of natural abundance, environmental benignity and similar intercalation chemistry with lithium^3^. Polyanion phosphate-based cathode materials, such as NaVOPO_4_F_0.5_, Na_2_FePO_4_F, Na_3_V_2_(PO_4_)_2_F and Na_3_V_2_(PO_4_)_2_F_3_, recently, have been received much attention due to their excellent structural, good thermal stabilities and strong networks^5–8^. A NASICON-type Na_3_V_2_(PO_4_)_3_ is one of the most potential cathode material for Na-ion storage. Na_3_V_2_(PO_4_)_3_ exhibits two distinct potential plateaus in the V^4+^/V^3+^ and V^3+^/V^2+^ redox couples at 3.4 V and 1.6 V vs. Na/Na^+^, corresponding to theoretical capacities of 117.6 and 58.8 mAh g^−1^, respectively^9^. Moreover, the pristine Na_3_V_2_(PO_4_)_3_ suffers from severe capacity fading at high rate because of the low electrical conductivity and poor ion diffusivity^10^. To improve rate capability and cycle life of Na_3_V_2_(PO_4_)_3_, much efforts have been made by different approaches, such as doping of other cation or anion, including K^+^, Al^3+^, Fe^3+^, Mn^2+^, Mg^2+^ or B^3+^ ^11–17^, carbon-coating using various carbon precursors and reducing the particle size via optimizing synthetic processes^18–23^. The substitution of other metal/non-metal in Na_3_V_2_(PO_4_)_3_ are, indeed, quite efficient way to enhance electrochemical properties compared to bare one. Additionally, some of cation substitutions are unstable or toxic, need to be strongly electronegative enough to reduce the valence of vanadium ion and electrochemically active at similar potential not to sacrifice the gravimetric capacity. Therefore, the fluorination replacing O^2−^ ions by F^−^ ions was a rational alternative. The fluorine-based polyanion frameworks could be enhanced the working potentials and larger iconicity of transient metal-F^−^ bond compared to metal-oxide^17^. It is noteworthy that the modification of polyanion groups by F^−^ and their electrochemical affection factors studies are still quite interesting of identification and development of battery materials.

In this study, Na_3-*x*_V_2_(PO_4-*x*_F_*x*_)_3_ (*x* = 0, 0.1, 0.15 and 0.3) polyanion cathode materials were synthesized via a simple sol-gel method and evaluate the electrochemical performance for sodium-ion storage. Interestingly, the optimal fluorine in NVP material is improved the conductivity and induce the catalytic activity of pristine NVP. In addition, the symmetric full-cell was constructed via optimal amount of F-doped NVP used for cathode and anode material. It delivers the outstanding ultra-long term cycle performance.

## Results and Discussion

**Figure 1:**
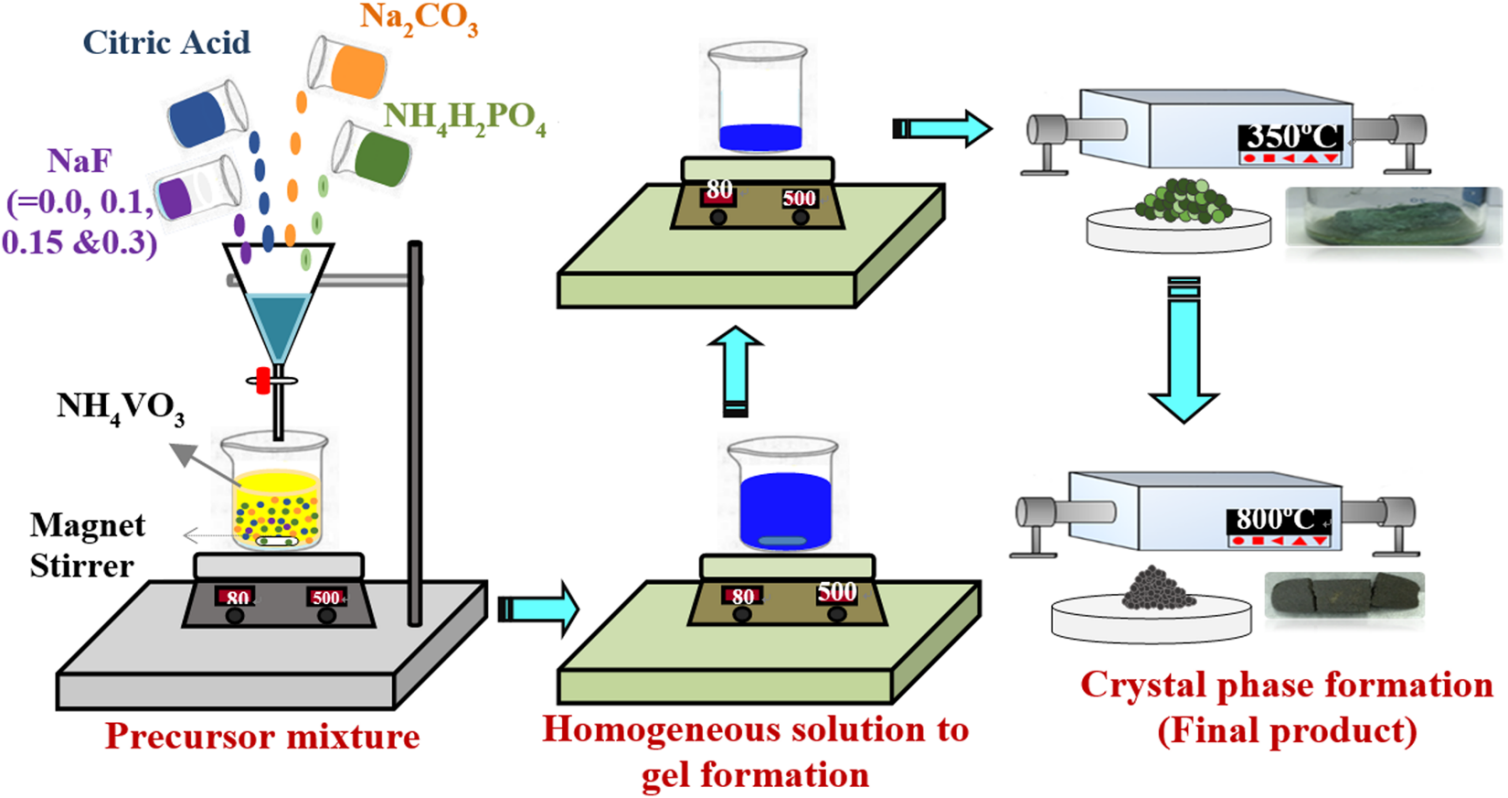
Schematic illustration of the Preparation of Na_3-*x*_V_2_(PO_4-*x*_F_*x*_)_3_ (*x* = 0.0, 0.10, 0.15 and 0.30) via Sol-Gel Method.

Figure 1 shows the schematic illustration of synthesize of NVP and F-doped NVP by a simple sol-gel technique. First, NH_4_VO_3_ was dissolved in distilled water under constant stirrer at 80 °C and yellow solution was observed. Then, citric acid was added drop by drop into the (VO_3_)^−^ aqueous solution and the color of solution was gradually changed from yellow to dark blue^24^. Afterwards, other precursors of Na, PO_4_ and F (for doping) were added into the solution. The mixed solution was constantly stirrer at 80 °C and formed as a homogeneous solution to viscous gel. Subsequently, as-prepared gel was dried in oven. Finally, sample was preheated at 350 °C for 4 h and calcined at 800 °C for 8 h in Ar. At the end, we could obtain a series of Na_3-*x*_V_2_(PO_4-*x*_F_*x*_)_3_ powder samples.

**Figure 2:**
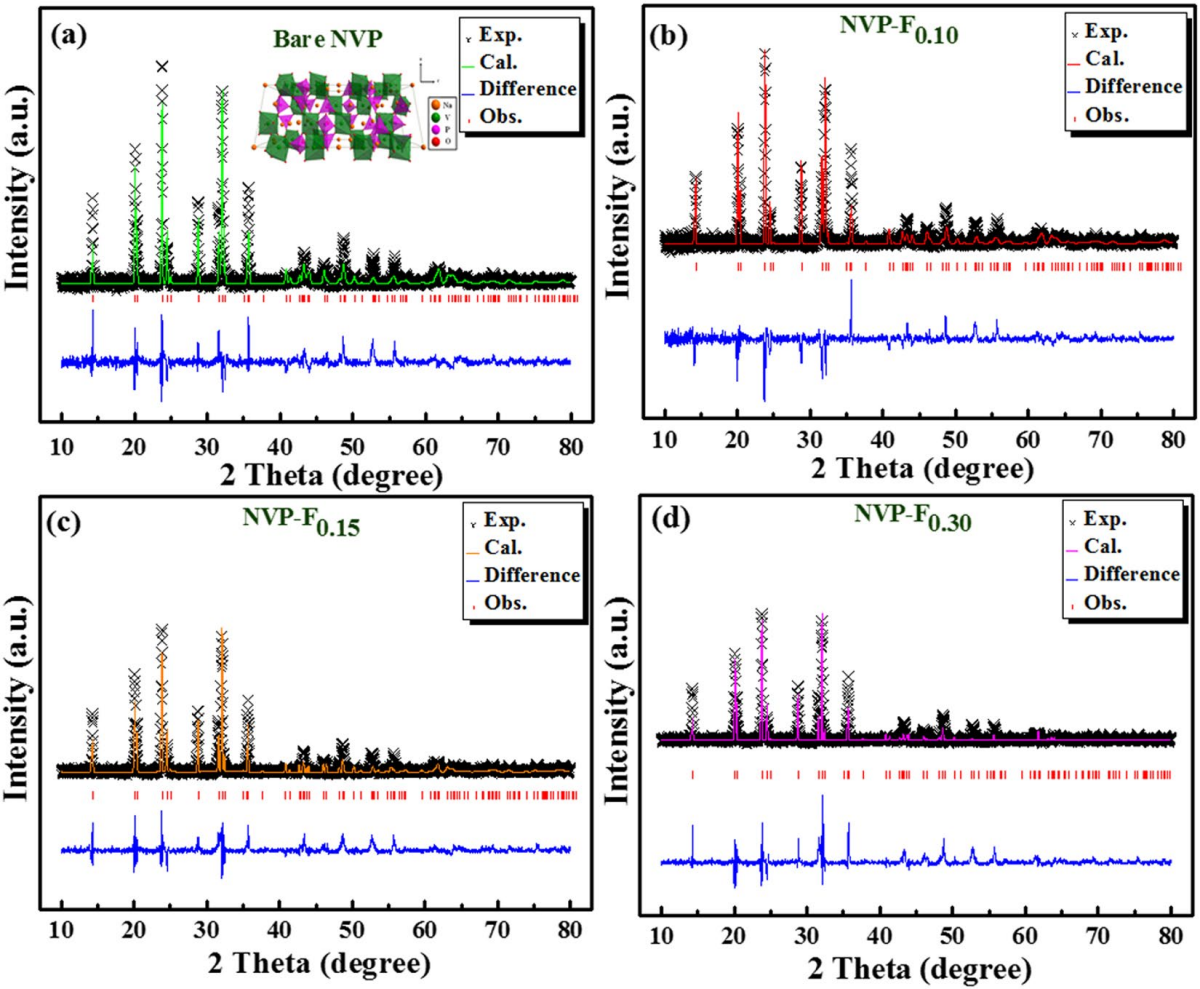
Rietveld refined XRD patterns of (**a**) bare NVP and (**b–d**) F-doped Na_3_V_2_(PO_4_)_3_, respectively.

Figure 2(a–d) represent the Rietveld refined PXRD patterns of bare and F-doped Na_3_V_2_(PO_4_)_3_ materials and the corresponding observed structural parameters are shown in Table S1. The diffraction peaks of prepared materials were related to the NASICON structure of rhombohedral phase with space group of *R-3c* ^25–27^. The refined unit cell lattice parameters are presented in Fig. S1a,b. The lattice parameters are epitomized that the values of ‘*a*’ axis (*a* = *b* axis) and the lattice constants of anionic (F^−^) atom doped NVP samples were slightly increased than pristine NVP. It may be related to the small amount of F-substitution with relatively smaller ionic radius (F^−^ = 1.33 Å) for O^2−^ (r = 1.40 Å) anion and larger than V^3+^ (0.64 Å)/V^2+^ (0.79 Å), Na^+^(1.02 Å) cations, respectively. Furthermore, the confirmation of F-doped sites was studied by Neutron powder diffraction analysis and the result of pristine and 0.15% F-doped NVP are shown in Fig. S2a and b, respectively.

According to the neutron powder diffraction result shows that the oxygen at two 36f sites was partially substituted by F-ions. Analysis of the occupancy factors shows that all sites are fully occupied except of sodium content is 5% deficient in F-doped sample. The chemical composition that we obtained from the fits of bare and 0.15 mol of F-doped sample are Na_3_V_2_(PO_4_)_3_ and Na_2.85_V_2_(PO_3.95_F_0.05_)_3_, respectively (Fig. S2a and b), which agrees very well with the stoichiometric composition. The neutron diffraction pattern refined structural parameters are summarized in Tables S2 and S3. Thus, the Neutron powder diffraction result represented that the fluorine substitution occupied in oxygen sites with a tiny deficiency in Na ion. The substitution of small ionic radius of fluorine anion was faintly increased the bare NVP volume (Fig. S1b). These results are coincident with other larger ionic radius metal ions doped NVP materials^11,27,28^.

**Figure 3:**
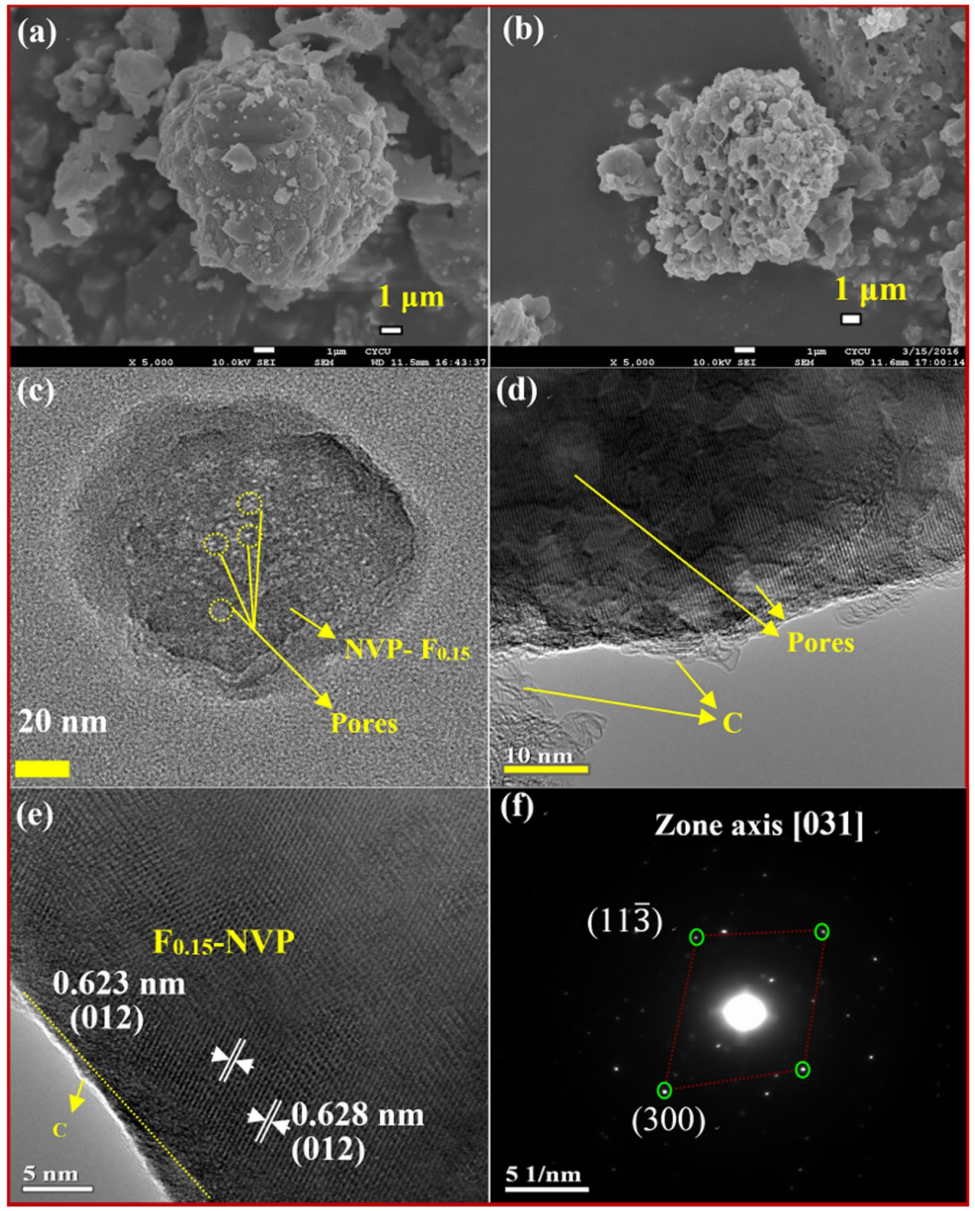
SEM image of (**a**) bare Na_3_V_2_(PO_4_)_3_, (**b**) NVP-F_*0*.*15*_ and HR-TEM images of NVP-F_*0*.*15*_, (**c**) low magnification, (**d**) high magnification, (**e**) lattice fringes image and (**f**) SAED pattern.

Figure 3a shows the bare NVP SEM image and 0.15% F-doped NVP SEM image is shown in Fig. 3b. The bare NVP sample was observed inhomogeneous agglomerated particles (Fig. 3a) and the NVP-F_*0*.*15*_ sample depicted more quantity of visible pores of the whole product (Fig. 3b). The calcination treatment to facilitate the degradation of the organic and fluoro-carbon groups also promotes the gas release, so as to engender this porous architecture of framework^29^. The morphology of NVP-F_*0*.*10*_ and NVP-F_*0*.*30*_ samples are shown in Fig. S3a and S3b. Figure 3c displays the HR-TEM image of synthesized NVP-F_*0*.*15*_ sample. It represented the agglomerate inhomogeneous morphology and the particle size was around 120 nm with porous nature. The porous can be clearly seen in Fig. 3d on the surface of prepared NVP-F_*0*.*15*_. Figure 3e shows the lattice fringes with d-spacing of 0.62 nm correspond to the (012) plane of NASICON-type NVP-F_*0*.*15*_.

Figure 3f shows the selected area electron diffraction (SAED) pattern of 0.15% F-doped NVP sample and can be indexed to the [031] zone axis^18^. The present result represents the good crystalline characteristics and consensus to XRD result. The EDX mapping images of bare and NVP-F_*0*.*15*_ are shown in Figs S4(a–f) and S5(a–f), respectively. Herein, all the elements presences are confirmed and uniformly distributed in the prepared samples. Fig. S6a demonstrates the wide-range core XPS spectra of bare and NVP-F_*0*.*15*_ samples. The results showed furthermore confirmation of the elements in prepared samples. The binding energies at 516.8 and 523.8 eV were attributed to the V^3+^ oxidation state of V 2p_3/2_ and V 2p_1/2_ on the prepared both samples (Fig. S6b). This was good agreement with the previously reports^26,30,31^. The binding energy of F 1s was observed at 684.15 eV for NVP-F_*0*.*15*_ sample (Fig. S6c). The fluorine substitution did not alter the valence states of bare material elements. The binding energies were observed around 1071.23, 133.36 and 531.31 eV corresponding to the Na^+^ 1s, P^+5^ 2p and O^2−^ 1s of synthesized both samples, respectively (Fig. S6(d–f)).

The pore size distribution and specific surface area were characterized using N_2_ adsorption-desorption and the results of bare and NVP-F_*0*.*15*_ samples are shown in Fig. S7 and inset in Fig. S7. The specific surface area of bare NVP and NVP-F_*0*.*15*_ were measured by Brunauer–Emmett–Teller (BET) method. The surface area of NVP and F-doped NVP were measured to be 8.3 m^2^ g^−1^ and 8.8 m^2^ g^−1^, respectively. The average pore size was found to be 25.64 and 15.68 nm of bare and NVP-F_*0*.*15*_ sample using Barrett-Joyner-Halenda (BJH) method. The results show that both specific surface area and pore size distribution of NVP w/wo doping were similar^22,32,33^.

**Figure 4:**
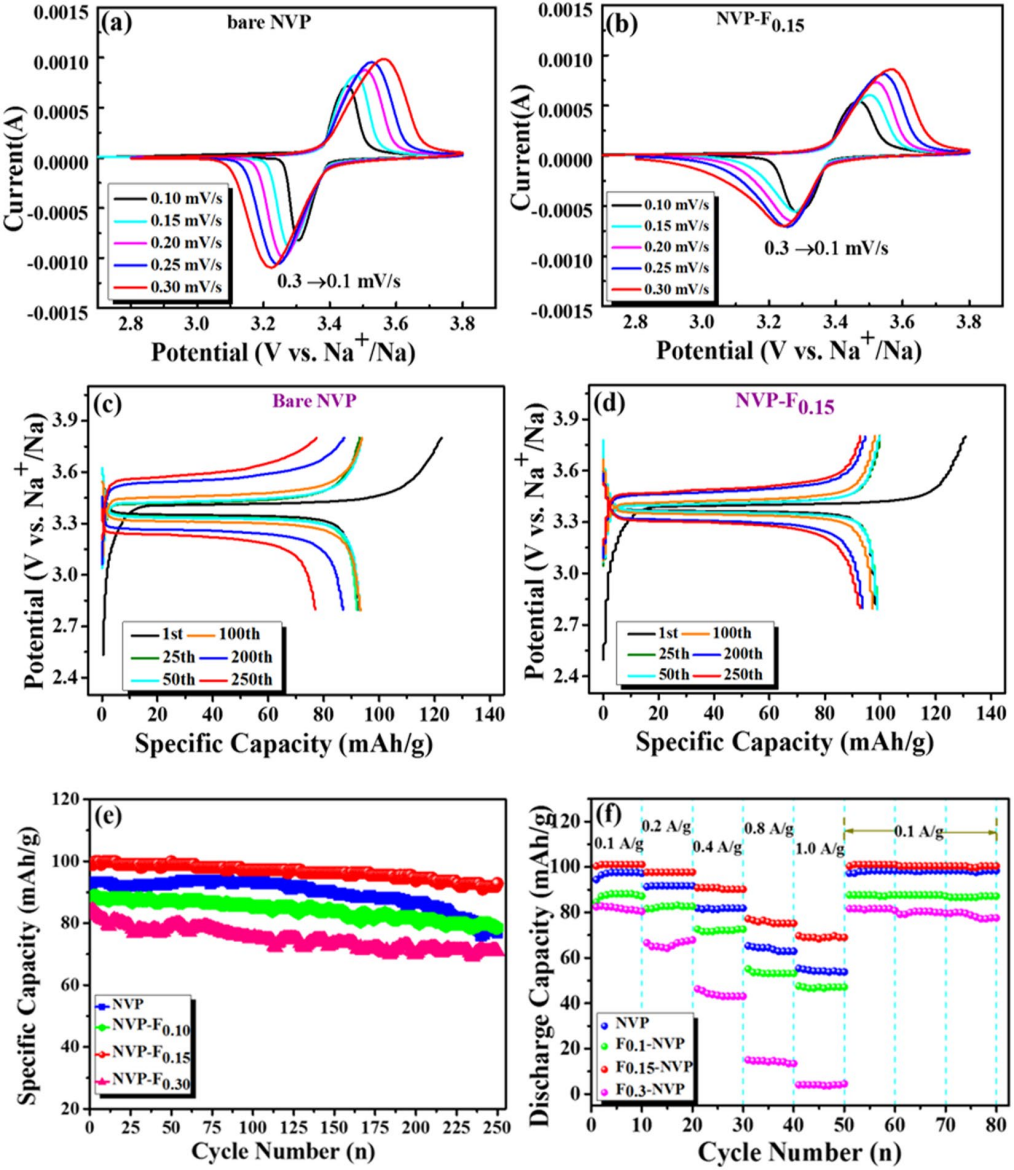
Electrochemical performance of the bare and F-doped NVP as cathode materials for half-cell configuration: (**a** and **b**) CV curves at different scan rates of NVP and NVP-F_*0*.*15*_ in the voltage range of 2.8–3.8 V vs. Na^+^/Na: different cycle charge/discharge profiles of (**c**) NVP and (**d**) NVP-F_*0*.*15*_ at 0.1 A g^−1^; (**e**) Cycle life tests of prepared bare and doped electrodes at 0.1 A g^−1^ for 250 cycles and (**f**) Rate capabilities at different current density.

The conductivity of active material is very important to Na-ion intercalation/de-intercalation processes. The electronic conductivity of as-synthesized samples was measured by using four-probes DC method in room temperature. The corresponding result is shown in Fig. S8. The conductivity of bare Na_3_V_2_(PO_4_)_3_ and Na_2.9_V_2_(PO_3.967_F_0.033_)_3_, Na_2.85_V_2_(PO_3.95_F_0.05_)_3_, Na_2.7_V_2_(PO_3.9_F_0.10_)_3_ were determined to be 1.63 × 10^−6^, 2.95 × 10^−6^, 6.33 × 10^−5^ and 1.67 × 10^−−6^ S/cm, respectively. Among these samples, the Na_2.85_V_2_(PO_3.95_F_0.05_)_3_ demonstrated better electrical conductivity. Fig. S9a and b show the cyclic voltammograms (CVs) of initial three cycles of bare and NVP-F_*0*.*15*_ electrodes at a scan rate of 0.1 mV s^−1^. The oxidation (Na extraction) and reduction (Na insertion) peaks were located at 3.45 V and 3.30 V for NVP and NVP-F_*0*.*15*_ electrode materials, respectively. The average voltage (ΔE) was observed at 3.37 V for both electrodes. It is close to the equilibrium voltage of V^4+^/V^3+^ redox couple in NVP^26^. It designates that the deintercalation/intercalation of two sodium-ions located at the M_2_ sites into a single formula unit of the prepared electrodes^26^. The potential difference (ΔV) was 0.15 V for NVP and 0.16 V for NVP-F_*0*.*15*_ electrodes. CV curves illustrate noticeably anodic peaks move to a lower potential, while the cathodic peaks slightly shift to higher potential, as a result indicates the inductive effect characteristic of NVP^34,35^. The diffusion coefficient of sodium-ion in NVP could be calculated from the linear relationship between their peak currents (*i*_p_) and the square root of scan rates (*v* ^1/2^) using the following Randles-Sevcik equation via CV analyses:^26,36,37^.

$${i}_{{\bf{p}}}/{\boldsymbol{m}}={\bf{0}}.{\bf{4463}}{({{\boldsymbol{F}}}^{{\bf{3}}}/{\boldsymbol{RT}})}^{{\bf{1}}/{\bf{2}}}{{\boldsymbol{n}}}^{{\bf{3}}/{\bf{2}}}{\boldsymbol{A}}{{\boldsymbol{D}}}^{{\bf{1}}/{\bf{2}}}{\boldsymbol{C}}{{\boldsymbol{v}}}^{{\bf{1}}/{\bf{2}}}$$

where, *i*_p_ is the peak current (A), *m* is the mass of the active cathode material, *F* is Faraday constant, *R* is gas constant, *T* is absolute temperature, *n* is number of electrons involved in the reaction (*n* = 2), *A* is the effective contact area between the electrode and the electrolyte, as obtained from the cathode material multiplied with the active mass ratio, and *C* is the concentration of Na ions in the cathode, as calculated from the crystallographic cell parameters of NVP. CV curves of bare NVP and NVP-F_*0*.*15*_ electrodes with different scan rate are shown in Fig. 4a,b. The corresponding relationship between the square root of the scan rate (*v* ^1/2^) and peak current (*i*_*p*_) are shown in Figure S10a and S10b. The calculated diffusion coefficient (D) of bare NVP and NVP-F_*0*.*15*_ samples were 5.68 × 10^−10^ cm^2^ s^−1^ and 6.37 × 10^−10^ cm^2^ s^−1^ for anodic and 6.03 × 10^−10^ cm^2^ s^−1^ and 6.46 × 10^−10^ cm^2^ s^−1^ for cathodic reaction, respectively. These results are in consistent with NVP reports^36,37^.

EIS was further used to investigate the electrochemical kinetics behaviour of fresh bare NVP and NVP-F_*0*.*15*_ electrodes. The Nyquist plot with corresponding fitted equivalent circuit model is presented in Fig. S11 and inset in Fig. S11. From the equivalent circuit, R_s_ is the electrolyte resistance and CPE represents the double layer capacitance and capacity of the surface layer. R_ct_ is the charge transfer resistance. The Warburg impedance (W) represents the diffusion behaviour at low frequencies^32^. The semicircle portion of the impedance plot defines the polarization of the electrode-electrolyte interface, which became smaller after F-doped into NVP. The charge transfer resistance of NVP-F_*0*.*15*_ (52 Ω) electrode was lower than that of bare NVP (276 Ω). Thus, by using F-doping in NVP material, charge transfer and ion transport kinetics could be effectively improved. In addition, An Exchange current density (i^0^) is one of the main factors to affect the kinetic process, which can be used to measure the catalytic activity of electrodes. The following equation describes the calculation of Exchange current density (i^0^)^38^:

$${{\bf{i}}}^{{\bf{0}}}={\bf{R}}{\bf{T}}/{\bf{n}}{\bf{F}}{{\bf{R}}}_{{\bf{c}}{\bf{t}}}$$

where R is the gas constant, T is the absolute temperature, n is the number of electrons per molecule during oxidization (n = 2) and F is the Faraday constant. The calculated exchange current density (i^0^) values were 0.0465 and 0.2470 mAh g^−1^ for bare and NVP-F_*0*.*15*_ samples, respectively. This result represents the F-doping to induce the catalytic activity of bare NVP.

Figure 4c,d depict the charge/discharge profiles of prepared bare and NVP-F_*0*.*15*_ electrodes at 1^st^, 25^th^,50^th^, 100^th^, 200^th^ and 250^th^ cycles in the current of 0.1 A g^−1^. The voltage curve has a flat charge plateau at about 3.4 V (0.1 A g^−1^), which corresponds to the redox of V^4+^/V^3+^ couple. This result is good agreement of CV results^26,39^. The initial discharge capacity of NVP and NVP-F_*0*.*15*_ electrodes were 98 mAh/g and 103 mAh/g, respectively (Fig. 4c,d). However, the reversible capacity of lower (NVP-F_*0*.*10*_) and higher (NVP-F_*0*.*30*_) content of fluorine doped NVP electrodes exhibited capacity of 85 and 82 mAh/g, as shown in Fig. S12a and b. The corresponding cyclic performance is presented in Fig. 4e. The discharge capacities of 78, 81, 96 and 73 mAh/g were observed at 250 cycles of NVP, NVP-F_*0*.*10*_, NVP-F_*0*.*15*_ and NVP-F_*0*.*30*_, respectively (Fig. 4e). The corresponding capacity retention was measured about 83, 89, 93 and 84% for NVP, NVP-F_*0*.*10*_, NVP-F_*0*.*15*_ and NVP-F_*0*.*30*_, respectively (Fig. S12(c–f)). The coulombic efficiency in the first cycle is very important for the application of Na-ion batteries, which is also the main barrier for the anode material^40^. The coulombic efficiencies of NVP, NVP-F_*0*.*10*_, NVP-F_*0*.*15*_ and NVP-F_*0*.*30*_ samples in the first cycle were 75.52, 72.01, 75.70 and 76.86%, respectively. (Shown in Fig. S12(c and f)). The lower coulombic efficiencies might be ascribed the following reasons: (i) The kinetic barriers by structural changes during the Na extraction;^41,42^ (ii) The activation process for the cell component during the electrochemical reactions to fluctuates the coulombic efficiency;^43^ (iii) The electrolyte decomposition and side reactions of PC based electrolyte systems^43,44^ and (iv) The dissolution of oxidized NVP for that the reaction of NVP can be fully ensued before the occurrence of oxygen evolution^45^. Moreover, the optimization of the suitable electrolyte is an effective vital role of coulombic efficiency improvement in SIBs^42,44,46^. The coulombic efficiency was observed 99% after initial few cycles and it was maintained upto 250 cycles of bare NVP and F-doped NVP electrodes (Fig. S12(c–f)). The F-doped NVP electrodes were observed more stable narrow charge-discharge polarization curves than bare NVP (Fig. 4d). It is endorsed to improve the conductivity of bare NVP by F-doping and induced the redox kinetics. The NVP-F_*0*.*15*_ electrode cell exhibits little higher capacity than prepared other electrodes and pervious reports of NVP^47–49^. Fig. 4(f) shows the rate capability of NVP and F-doped NVP electrodes at current density increasing from 0.1 to 1.0 A/g. Among these electrodes, NVP-F_*0*.*15*_ electrode cell exhibited more stable and remarkable rate performance. The stable discharge capacities of 103, 98, 91, 76 and 69 mAh g^−1^ were observed at 0.1, 0.2, 0.4, 0.8 and 1.0 A g^−1^, respectively, upto 5 cycles (98, 92, 82, 63, 55 and 97 mAh g^−1^ for bare NVP). After these C-rate tests, the discharge capacity of NVP-F_*0*.*15*_ sample maintained around 102 mAh g^−1^ at 0.1 A g^−1^ over the 30 cycles. The capacity retention was observed 98% with more stable cyclic performance at higher C rate of 1 A g^−1^.

**Figure 5:**
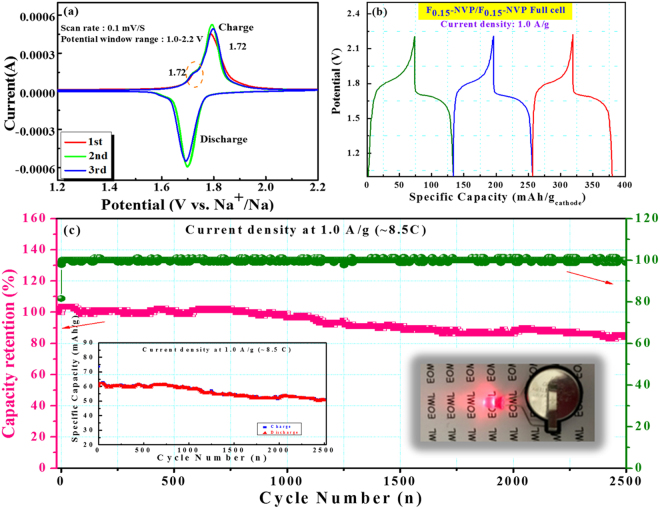
Electrochemical performance of the symmetric sodium-ion full-cell employing the NVP-F_*0*.*15*_ as both anode and cathode material (**a**) CV curves at a scan rate of 0.1 mV s^−1^ (**b**) Potential-capacity profile of first three cycles at 1.0 A g^−1^ in a voltage range of 1.0–2.2 V, and (**c**) capacity retention, efficiency and the corresponding inset of left side capacity curve at 1.0 A g^−1^ over 2500 cycles and right side display of LED using NVP-F_*0*.*15*_ electrodes constructed full-cell.

To approach practical application, symmetric full-cell fabricated by using NVP-F_*0*.*15*_ as cathode and anode materials was carried out in our study. Figure 5a illustrates CV curves at the first three cycles in potential window of 1.0–2.2 V with a scan rate of 0.1 mV s^−1^. The oxidation peaks were observed 1.72 V and 1.78 V and the reduction peaks were 1.68 and 1.60 V, which were good agreement with the output voltage plateau of charge/discharge curve (V^3+^/V^2+^ for redox reactions 1.6 V) and earlier reports^26,49–51^. Fig. 5b shows the initial three potential-capacity profiles in the current density of 1.0 A g^−1^. The charge/discharge capacity was 74/60 mAh g^−1^ with retention about 98% over the 1000 cycles at 1.0 A g^−1^, respectively. The first three charge/discharge profiles revealed the voltage plateaus of charge/discharge were appeared at ~1.78 V and ~1.69 V, which were very close to the CV values and previously reports^26,32,49,50^. As shown in Fig. 5(c), it revealed that the capacity retention reached 83% with superior stability throughout 2500 cycles, while the coulombic efficiency reached almost 100% and it maintained upto 2500 cycles with slightly decay. The ~100% of coulombic efficiency can be ascribed to the nature of the polyanionic-based electrode material, especially NVP composite cathode material. Inset of left hand side in Fig. 5(c) shows the charge/discharge capacity at 1.0 A g^−1^ over the 2500 cycles and inset of right hand side in Fig. 5(c) represents the LED lighted by our symmetric full cell. These results demonstrate the outstanding long-term cycle stability of the NVP-F_*0*.*15*_//NVP-F_*0*.*15*_ symmetric full-cell.

The excellent electrochemical performance of NVP-F_*0*.*15*_ electrode for both half and full-cells could be ascribed to (i) the higher electronic conductivity, (ii) improves the kinetics and catalytic behaviour of bare NVP, (iii) the slight lattice alteration of NASICON structure permits a fast transport of Na ions. Besides, the transition metal of V provides a wide range of charge compensation during the Na^+^ intercalation process. These are favourable to enhance the ionic conductivity. The long-terms cycling stability owing to the protective surface films on the electrode materials were formed in the PC based electrolyte system, which was beneficial for the improved passivation and suppression of side reactions between the Na metal and the PC based electrolyte solution containing Na salts^40^.

## Conclusions

A series of NASICON-type F-doped sodium vanadium phosphate polyanion materials were successfully synthesized by simple sol-gel method and the results were compared with bare material. The rhombohedral crystalline phase of both samples was observed by X-ray diffraction techniques. The lattice parameters of F-doped samples were slightly changed, which represented the small amount of substitution on the host material. The neutron powder diffraction results demonstrated that the oxygen at two 36f sites was partially substituted by F-ions with 5% of sodium content deficiency in the F-doped sample. The presences of fluorine and vanadium state were confirmed by XPS. The optimal F-doped NVP (NVP-F_*0*.*15*_) sample was observed porous morphology, superior conductivity and better catalytic effect than bare NVP. In half-cell Na-ion storage, the NVP-F_*0*.*15*_ electrode exhibited higher capacity, good cycle life and high rate capability than rest materials. It was observed initial discharge capacity of 103 mAh g^−1^ and bare NVP electrode was 98 mAh g^−1^ at 0.1 A/g. The symmetric Na-ion full-cell was fabricated using NVP-F_*0*.*15*_ as both cathode and anode material. The charge/discharge capacity was observed 74/60 mAh/g with retention about 98% over the 1000 cycles. Afterwards, the retention was reduced to 83% throughout the 2500 cycles and 100% of coulombic efficiency was maintained at 1.0 A/g, respectively. Overall this investigation, the superior appealing features of optimal F-doped NVP dual electrode material, such as high rate capability and long-term cyclic stability, will qualify their utilization in the advanced large-scale low cost energy storage applications.

## Methods

### Materials synthesis

Sodium carbonate (Na_2_CO_3_, Aldrich, 99.99%), ammonium vanadate (NH_4_VO_3_, Alfa-Acer, 99.99%), ammonium dihydrogen phosphate (NH_4_H_2_PO_4_, Aldrich, 99.99%), sodium fluoride (NaF, Aldrich, 99.99%) and citric acid (Aldrich, 99.99%) were used as precursor materials. Sol-gel method was used to synthesize a sequence of Na_3-*x*_V_2_(PO_4-*x*_F_*x*_)_3_, where *x* = 0, 0.1, 0.15 and 0.3 (as shown in Fig. 1). To synthesize Na_3-*x*_V_2_(PO_4-*x*_F_*x*_)_3_ materials, NH_4_VO_3_ was dissolved first into distilled water and then the solution was stirred at 80 °C. After a clear solution formed, appropriate amount of citric acid, NH_4_H_2_PO_4_, Na_2_CO_3_ and NaF solutions were drop by drop added into the NH_4_VO_3_ solution one by one with vigorously stirring at 80 °C. The gel was formed after several hours. The obtained gel was ground in a mortar and preheated at 350 °C for 4 h. Then, the powder was again ground and calcined at 800 °C for 8 h in flowing argon atmosphere. Finally, we can obtain F-doped Na_3_V_2_(PO_4_)_3_ powder. For comparison, bare NVP sample (No NaF adding) was synthesized by the same processes. Na_3-*x*_V_2_(PO_4-*x*_F_*x*_)_3_ (*x* = 0, 0.1, 0.15, 0.3) are represented as bare NVP, NVP-F_*0*.*10*_, NVP -F_*0*.*15*_ and NVP -F_*0*.*30*_, respectively.

### Characterization

The crystal structure and phase purity of a series of Na_3-*x*_V_2_(PO_4-*x*_F_*x*_)_3_ samples were studied by powder X-ray powder diffraction (XRD) using a Bruker D8 diffractometer with monochromatic Cu Kα radiation (λ = 1.54060 Å) operated at 40 KV and 30 mA. The powder XRD data were collected in a 2θ range from 10° to 80°. Neutron powder diffraction (NPD) data were collected on the high resolution powder diffractometer Echidna at the OPAL facility (Lucas Heights, Australia) using neutrons of wavelength 2.4395 Å selected from the Ge (331) monochromator^52^. The two powder samples were loaded into the standard 9-mm vanadium cans, and data were collected at room temperature. A vacuum chamber was used for reducing the air scattering. Both XRD and neutron diffraction patterns were analyzed using the General Structure Analysis System (GSAS) program, following the Rietveld refinement method. The morphological observation of prepared materials were characterized via field emission scanning electron microscopy (FESEM, JSM-7600F, JEOL) with energy dispersive spectrometer/electron mapping (EDS, X-MAX) and high-resolution transmission electron microscopic (HR-TEM) (Techni G2 S-TWIN, FEI) technique. Chemical valence states of the elements were investigated by X-ray photoelectron spectroscopy (XPS, PHI model 5802). A Tristar 3000 accelerated surface area and porosimetry instrument was used to measure the prepared products N_2_ adsorption/desorption isotherms.

### Electrochemical Measurement

A half-cell assembly of Na-metal | NaClO_4_: PC | NVP (or NVP-F) and symmetric full-cell of NVP-F_0.15_| NaClO_4_: PC | NVP-F_0.15_ were used to evaluate the electrochemical performance via CR2032 type coin cells. The cathodes (positive electrodes) were prepared by mixing of 70 wt. % of active material (NVP or NVP-F powder), 10 wt. % of Super P and 20 wt. % of Poly-(vinylidene fluoride) (PVdF, Kynar® HSV 900, Arkema Inc.) in N-methylpyrrolidone (NMP, ChromAR®, Macron Fine Chemicals TM) solvent to form a homogeneous slurry. Afterwards, the mixed slurry was spread uniformly on a thin aluminum foil and dried in vacuum at 120 °C for 6 h and then roll pressed. The samples were punched into circular discs. Glass fiber (Type A/E, P/N 61630, Pall Corporation) as separator was drenched in the electrolyte for 24 h prior to use. The coin cell assembling procedures were performed using Ar-filled glove box by keeping both the oxygen and moisture levels less than 1 ppm. The galvanostatic charge-discharge measurements were performed using a AcuTech battery testing system (Taiwan, R.O.C, model 750B) in the potential range of 2.8–3.8 V (V vs. Na^+^/Na) at ambient temperature with different C-rates. The cyclic voltammograms (CV) were measured by CH Instruments Analyzer CHI 6273E at a scan rate of 0.1 mVs^−1^ voltage window between 2.8 V and 3.8 V. The AC impedance was tested in the range from 0.1 Hz to 100 KHz with the amplitude of 5 mV. The symmetric Na-ion full-cell was fabricated using NVP-F_*0*.*15*_ as both electrode materials and evaluated the cycling performance. The cathode/anode weight ratio was maintained 1:2. The current density (A g^−1^) and capacity values (mAh g^−1^) presented throughout this study were calculated based on the total mass of the electrode materials.

## Electronic supplementary material

Supporting data
