# Supplementary material for: An Efficient Evaluation of F-doped Polyanion Cathode Materials with Long Cycle Life for Na-Ion Batteries Applications
Source: Sci Rep. 2017 Nov 1;7:14808. doi: 10.1038/s41598-017-13718-0 (PMC5665866; doi:10.1038/s41598-017-13718-0)
Supplement: Supplementary file 1 — Supporting data [file 41598_2017_13718_MOESM1_ESM.pdf]

Supporting Information

An Efficient Evaluation of F-doped Polyanion Cathode Materials with Long Cycle Life for Na-Ion Batteries Applications

Rasu Muruganantham,[1] Yi-Tang Chiu,[1] Chun-Chuen Yang,[2] Chin-Wei Wang[3] and Wei-Ren Liu[1,*]

[1] Department of Chemical Engineering, Chung Yuan Christian University, Taoyuan City, Chungli 32023, Taiwan, R.O.C.

[2] Department of Physics, Chung Yuan Christian University, Taoyuan City, Chungli 32023, Taiwan, R.O.C.

[3] Neutron Group, National Synchrotron Radiation Research Center, Hsinchu City, 30076, Taiwan, R.O.C.

**E-mail address: WRLiu1203@gmail.com*

Tel:+886 3-265-4140; fax: +886 3-265-4199

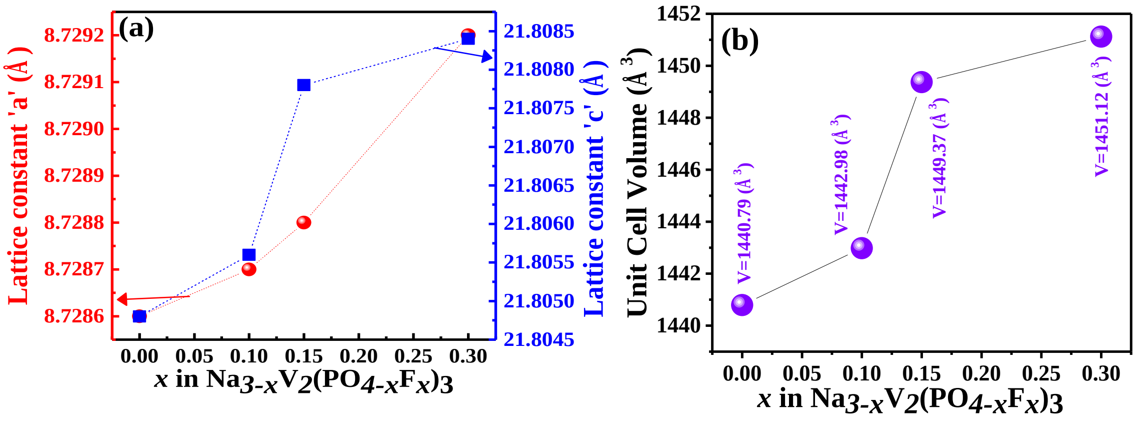

Figure S1. estimated lattice parameter values (**a**) lattice constant "*a*" & "*b*" and (**b**) cell volume.

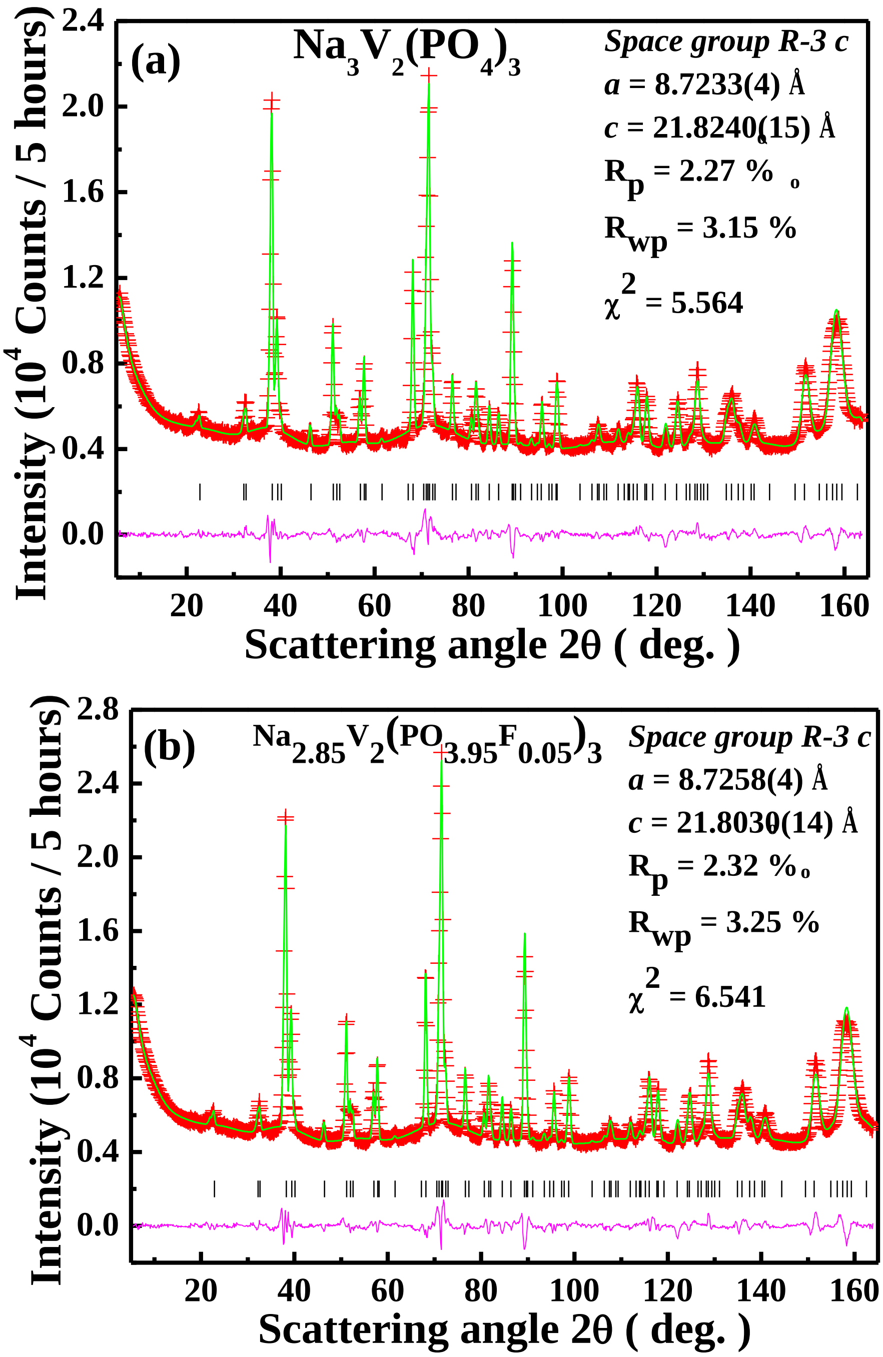

Figure S2. Neutron powder Diffraction patterns of (**a**) NVP and (**b**) NVP-$F_{0.15}$, respectively.

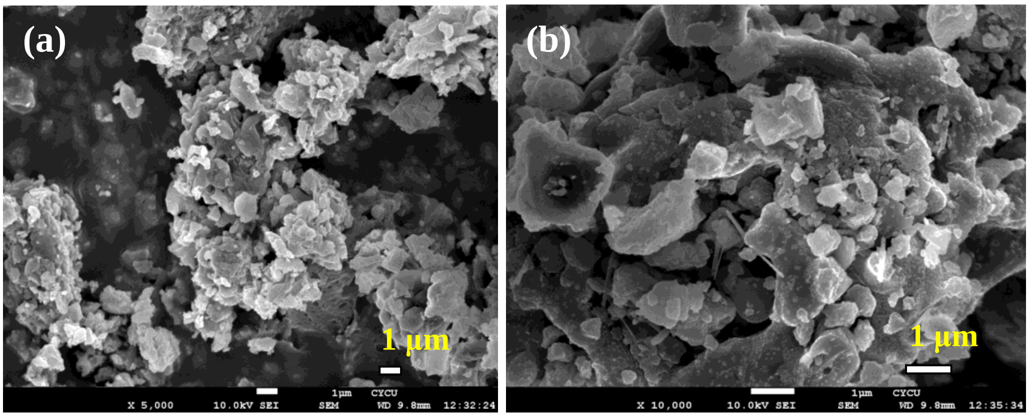

Figure S3. SEM image of (**a**) NVP-F$_{0.10}$ and (**b**) NVP-F$_{0.30}$.

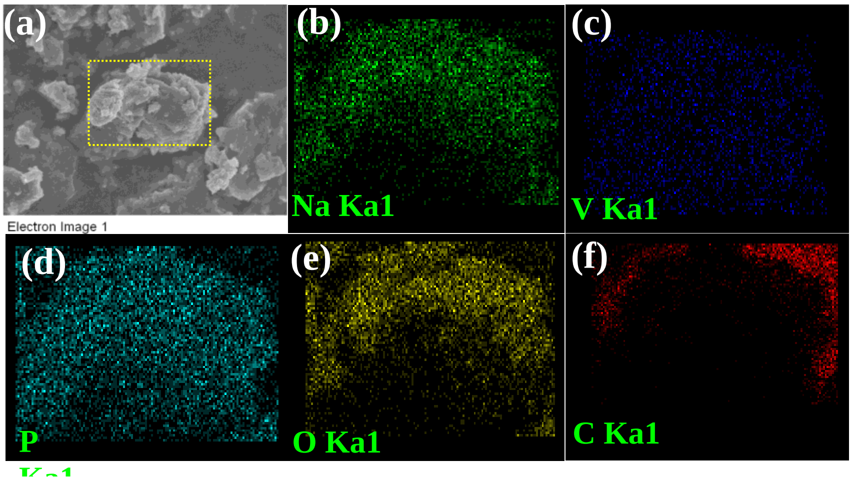

Figure S4. EDX mapping images of bare NVP (**a**) region selected for elemental mapping, (**b-f**) the corresponding EDX elemental mapping images of Na, V, P, O and C, respectively.

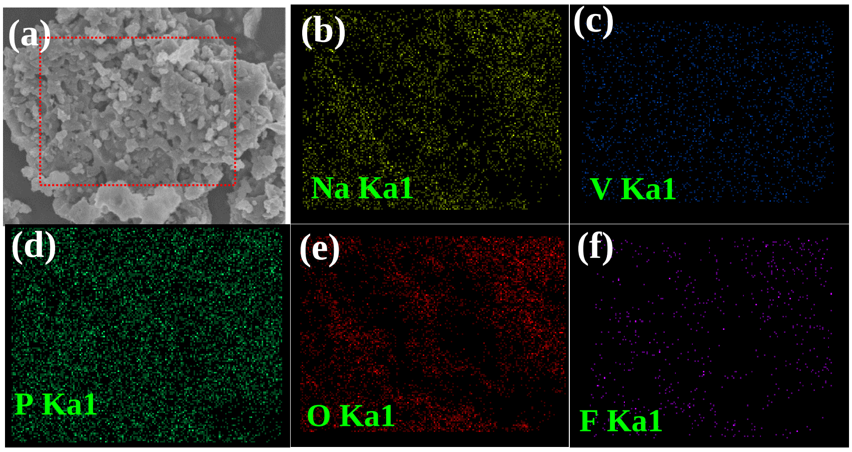

Figure S5. EDX mapping images of NVP-F$_{0.15}$ (**a**) region selected for elemental mapping, (**b-f**) the corresponding EDX elemental mapping images of Na, V, P, O and F respectively.

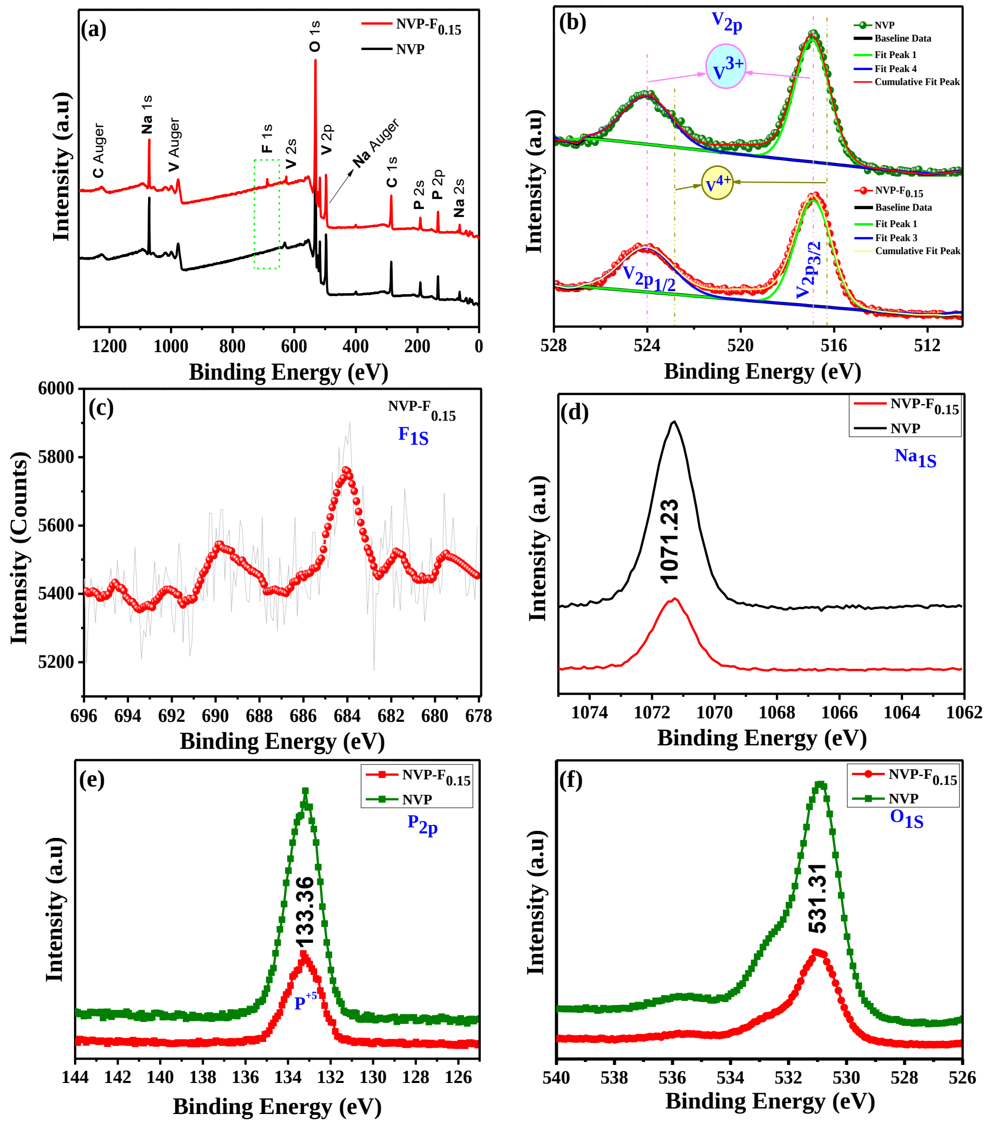

Figure S6. X-ray photoelectron spectra (XPS) of (**a**) wide range spectra, (**b**) V 2p core spectra of NVP and NVP-$F_{0.15}$, (**c**) F 1s core spectra of NVP-$F_{0.15}$ material and core spectra of (**d**) Na 1s, (**e**) P 2p and (**f**) O 1s, respectively.

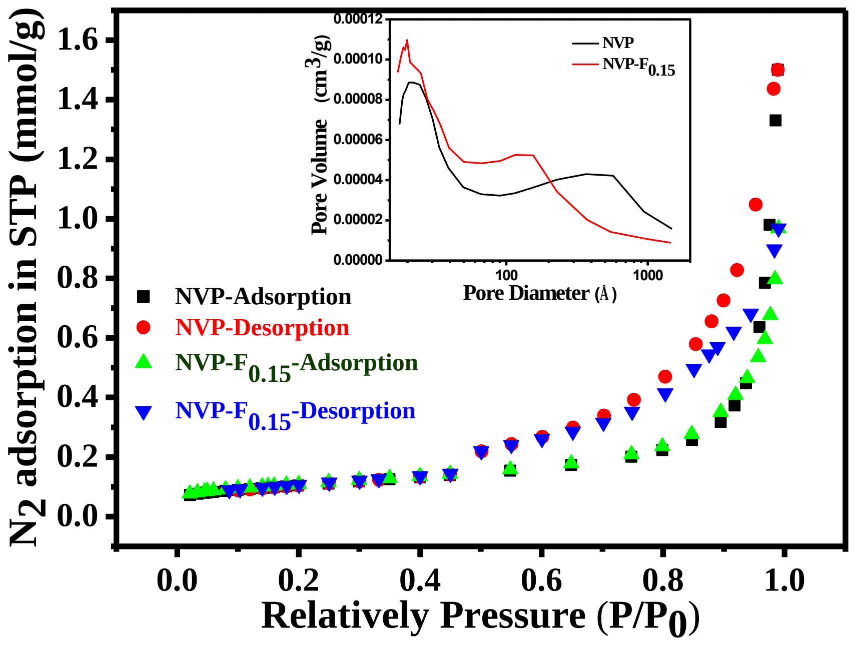

Figure S7. N_2 adsorption/desorption isotherm and the corresponding pore size distribution (the inset) of synthesized NVP and NVP-$F_{0.15}$ materials.

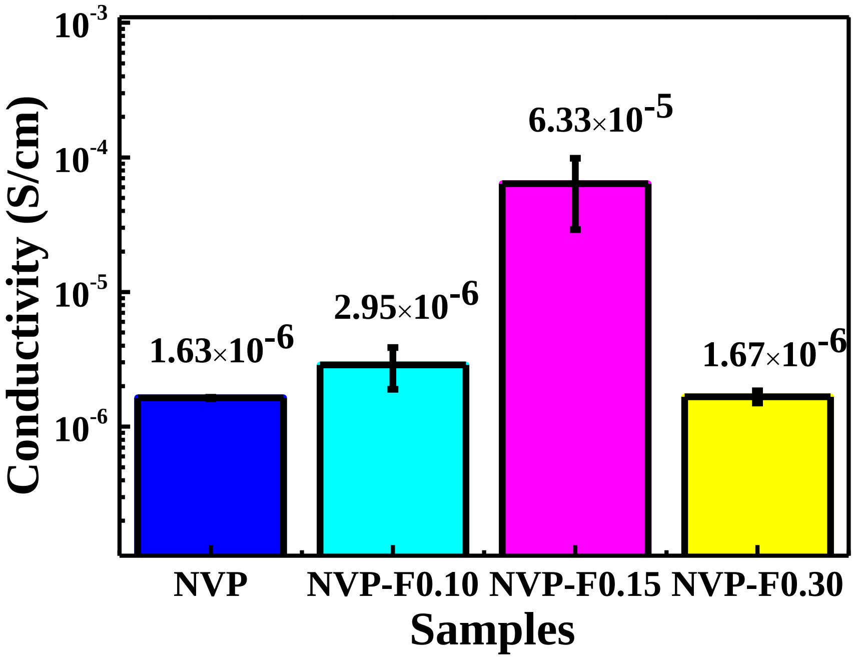

Figure S8. Electrical conductivity values of prepared both samples using four-probe method at room temperature.

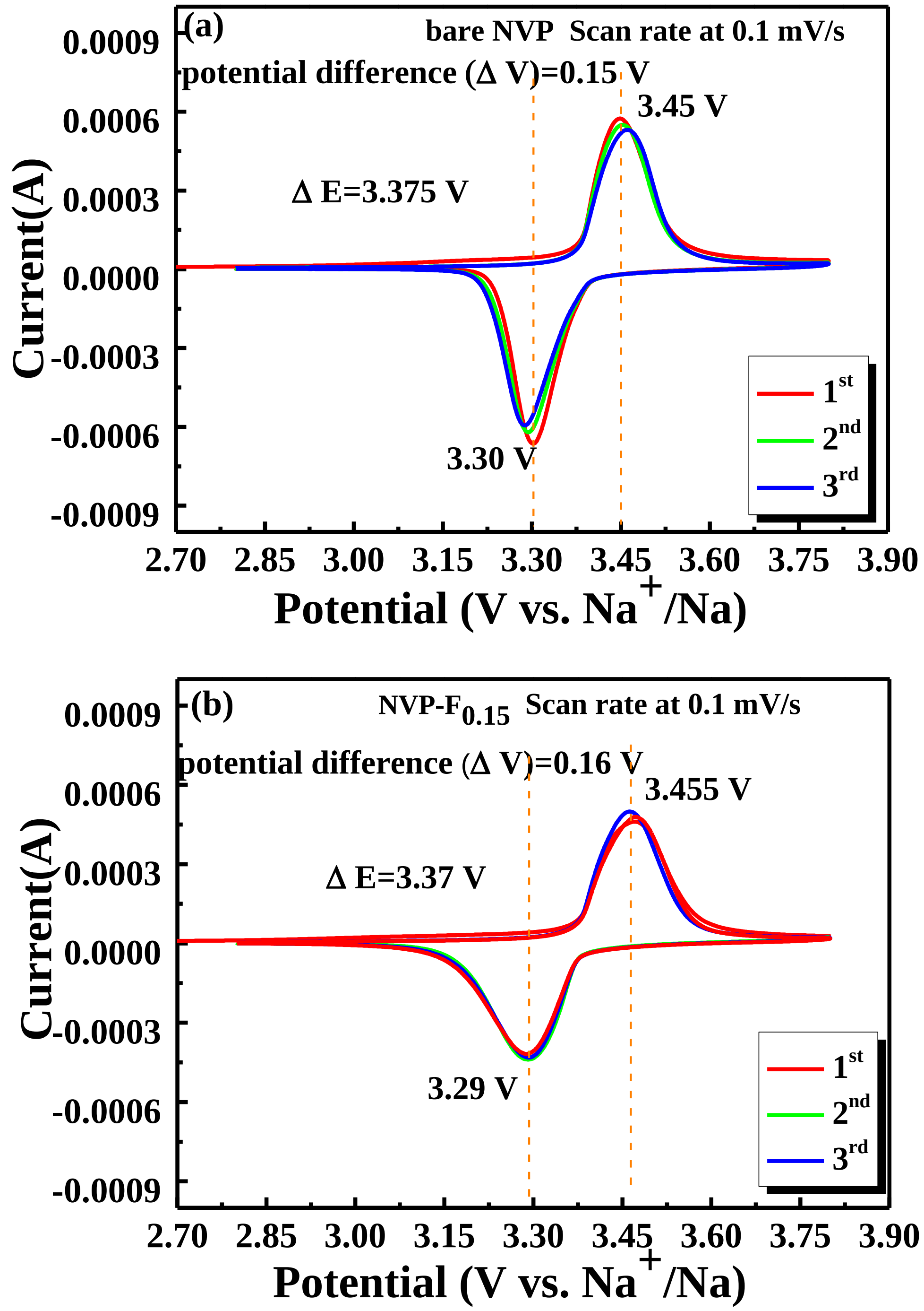

Figure S9. (**a & b**) bare NVP and NVP-$F_{0.15}$ electrode consisted half-cell CV curves of initial three cycles in the potential window of 2.8 to 3.8 V *vs.* Na^+/Na at a scan rate of 0.1 mV s^{-1}.

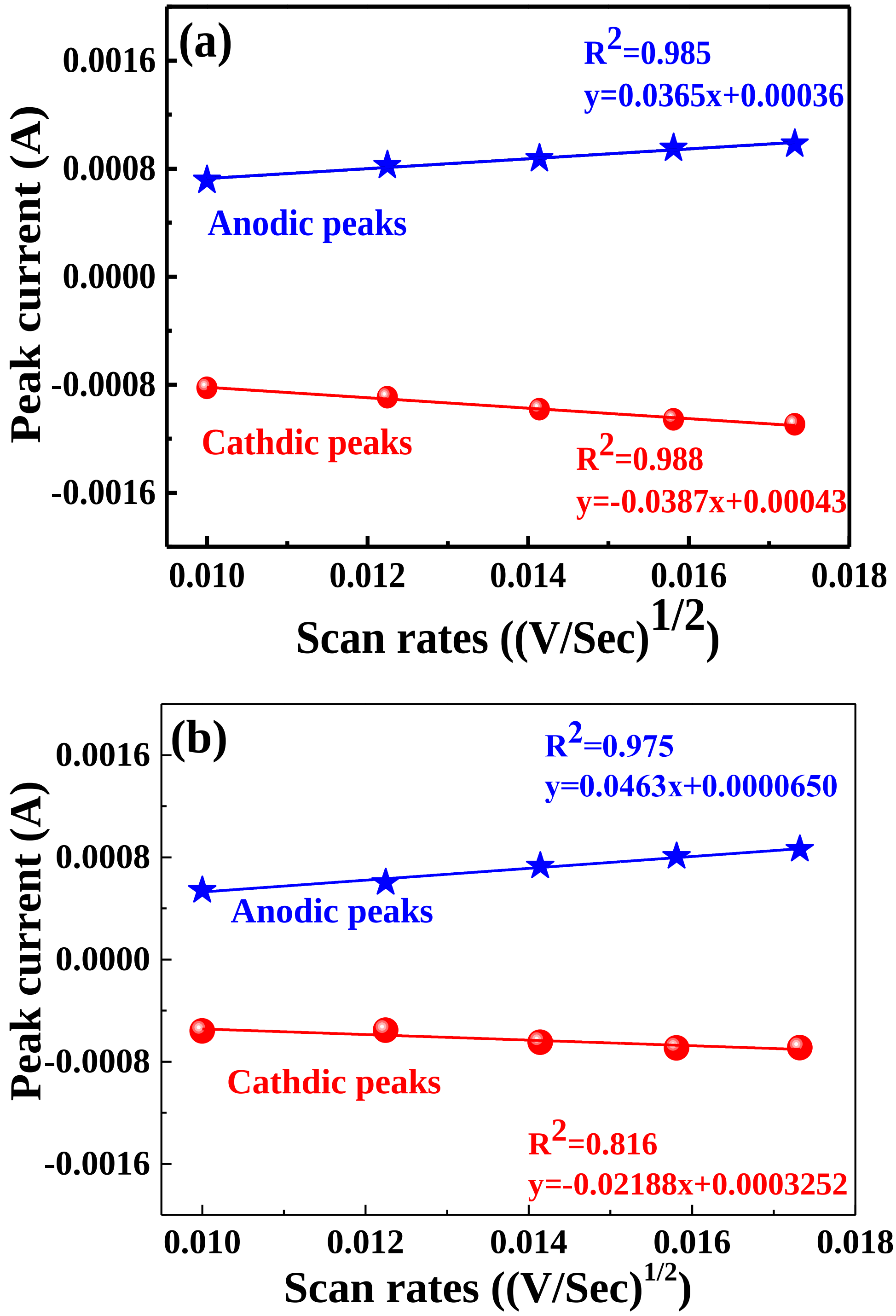

Figure S10. Linear fitting curve of the relationship between the square root of the scan rate $v^{1/2}$ and peak current i_p of (**a**) NVP and (**b**) NVP-F$_{0.15}$.

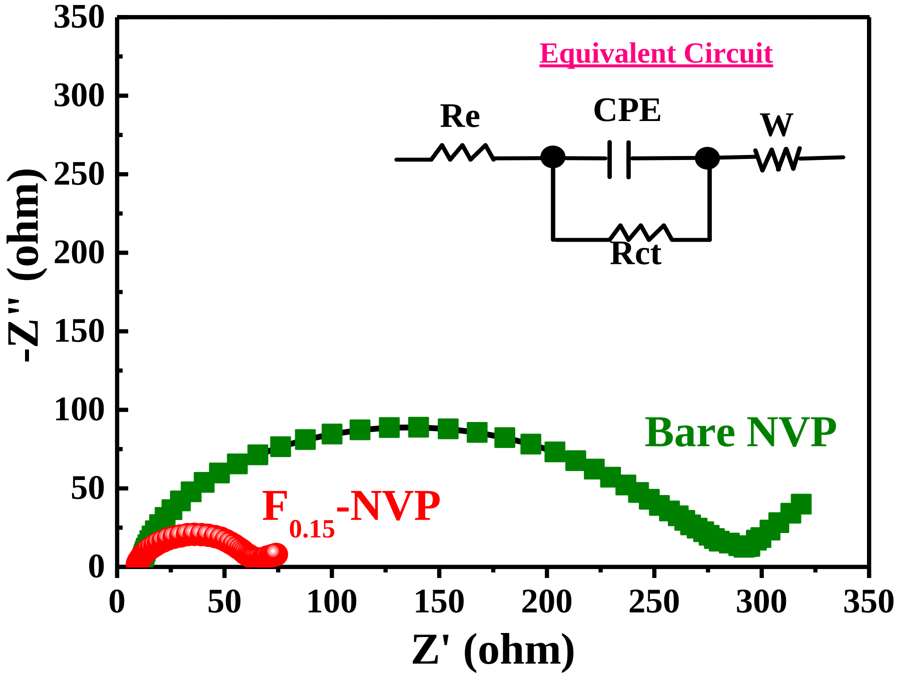

Figure S11. Nyquist plots of the NVP and NVP-$F_{0.15}$ in fresh state. Inset is a corresponding equivalent circuit.

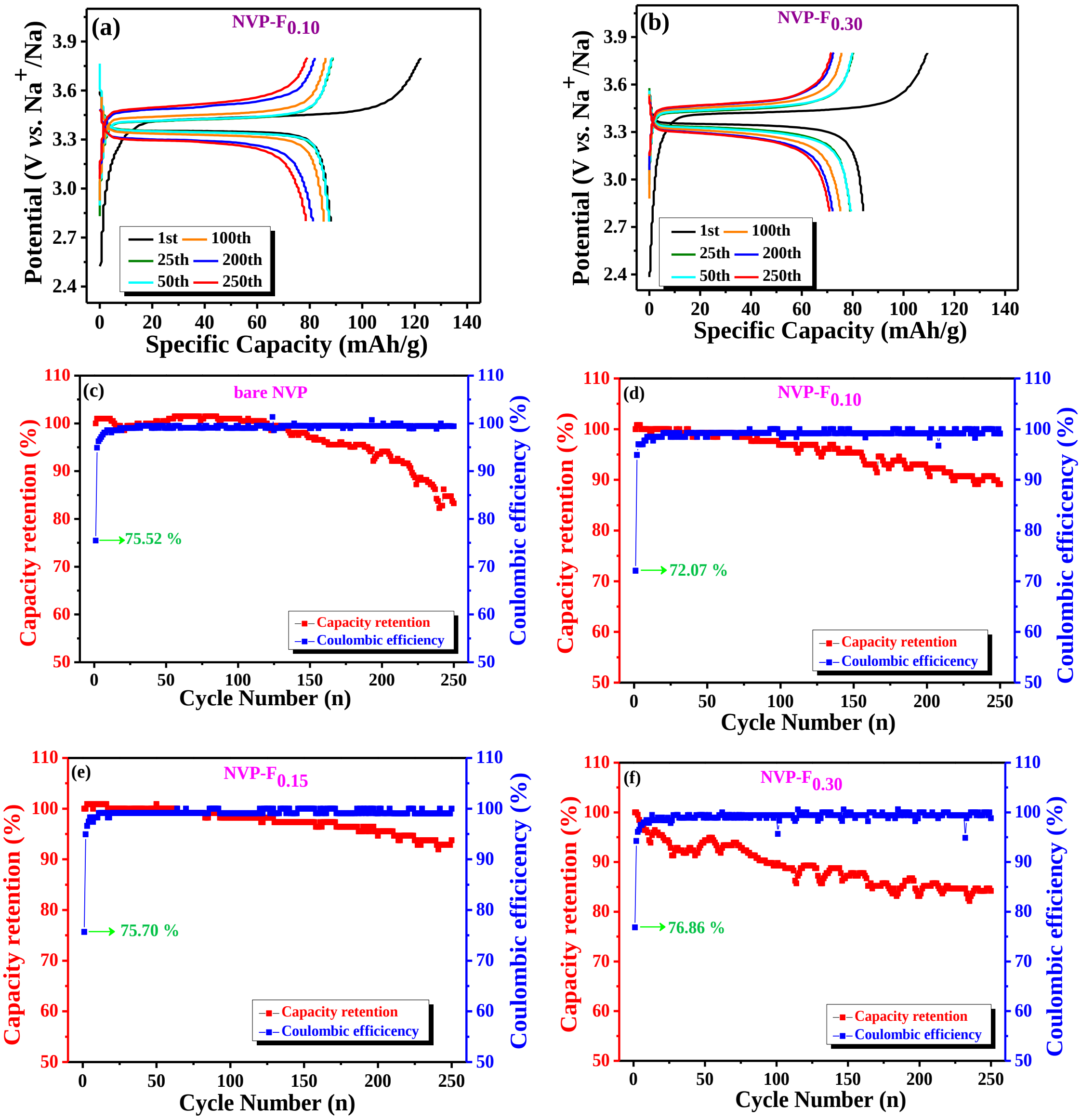

Figure S12. (a & b) charge/discharge profile of different cycles of NVP-$F_{0.10}$ and NVP-$F_{0.30}$ electrodes and cyclic performances at a current density of 0.1 A g^{-1} upto 250 cycles (**c**) bare NVP, (**d**) NVP-$F_{0.10}$ (**e**) NVP-$F_{0.15}$ and (**f**) NVP-$F_{0.30}$, respectively.

Table S1. represents the crystallographic data of prepared samples derived from rietveld refinements of powder XRD patterns.

Sample	*Atom*	X	Y	Z	Ui/Ue*100	Site sym	Mult	Type	Seq	Fractn
**Bare NVP**	*V1*	0.3333	0.6667	5.193(4)	8.0(28)	3	12	V+3	1	1
	Na1	0.3333	0.6667	0.1667	3.8(18)	-3	6	Na+1	2	0.805
	Na2	0.6667	0.9857(26)	0.0833	19.5(11)	2(010)	18	Na+1	3	0.731
	P1	-0.069(5)	0.3333	0.0833	80.0(31)	2(100)	18	P	4	1
	O1	0.8078(18)	0.9645(32)	0.0582(4)	80.0(23)	1	36	O-2	5	1
	O2	0.4485(29)	0.8245(12)	-0.0224(8)	80.0(20)	1	36	O-2	6	1

Sample	*Atom*	X	Y	Z	Ui/Ue*100	Site sym	Mult	Type	Seq	Fractn
**NVP-$F_{0.10}$**	*V1*	0.3333	0.6667	1.1118(24)	1.2(15)	3	12	V+3	1	1
	Na1	0.3333	0.6667	0.1667	0.9(6)	-3	6	Na+1	2	0.805
	Na2	0.6667	0.944(5)	0.0833	39.6(28)	2(010)	18	Na+1	3	0.731
	P1	-0.0091(22)	0.3333	0.0833	26.2(10)	2(100)	18	P	4	1
	O1	0.2640(53)	0.5065(26)	0.0941(5)	80.0(25)	1	36	O-2	5	1
	O2	0.4624(30)	0.8346(15)	-0.0091(7)	65.1(16)	1	36	O-2	6	1

Sample	*Atom*	X	Y	Z	Ui/Ue*100	Site sym	Mult	Type	Seq	Fractn
**NVP-$F_{0.15}$**	*V1*	0.3333	0.6667	2.1035(17)	-4.2(11)	3	12	V+3	1	1
	Na1	0.3333	0.6667	0.1667	1.4(6)	-3	6	Na+1	2	0.805
	Na2	0.6667	0.9705(17)	0.0833	10.5(7)	2(010)	18	Na+1	3	0.731
	P1	-0.025(4)	0.3333	0.0833	50.6(16)	2(100)	18	P	4	1
	O1	0.0179(30)	0.2017(27)	0.0907(9)	80.0(22)	1	36	O-2	5	1
	O2	0.4965(15)	0.8523(12)	-0.0127(8)	60.4(12)	1	36	O-2	6	1

Sample	*Atom*	X	Y	Z	Ui/Ue*100	Site sym	Mult	Type	Seq	Fractn
**NVP-$F_{0.30}$**	*V1*	0.3333	0.6667	-9.930(5)	-9.0(4)	3	12	V+3	1	1
	Na1	0.3333	0.6667	0.1667	-8.7(6)	-3	6	Na+1	2	0.805
	Na2	0.6667	4.956(7)	0.0833	10.2(23)	2(010)	18	Na+1	3	0.731
	P1	-0.743(7)	0.3333	0.0833	-9.0(30)	2(100)	18	P	4	1
	O1	-0.1161(20)	0.2811(18)	0.1904(6)	10.8(7)	1	36	O-2	5	1
	O2	0.3804(221)	0.7414(191)	-0.0042(5)	18.4(12)	1	36	O-2	6	1

samples	**NVP**			**NVP-$F_{0.10}$**			**NVP-$F_{0.15}$**			**NVP-$F_{0.30}$**		
Atom	*U11*	*U22*	*U33*	*U11*	*U22*	*U33*	*U11*	*U22*	*U33*	*U11*	*U22*	*U33*
V1	8.0(28)	8.01	8.01	*1.2(15)*	1.23	1.23	*-4.2(11)*	-4.22	-4.22	*-9.0(4)*	-9	-9
Na1	3.8(18)	3.79	3.79	*0.9(6)*	0.88	0.88	*1.4(6)*	1.43	1.43	*-8.7(6)*	-8.72	-8.72
Na2	19.5(11)	19.47	19.47	*39.6(28)*	39.56	39.56	*10.5(7)*	10.5	10.5	*10.2(23)*	10.24	10.24
P1	80.0(31)	80	80	*26.2(10)*	26.23	26.23	*50.6(16)*	50.63	50.63	*-9.0(30)*	-9	-9
O1	80.0(23)	80	80	*80.0(25)*	80	80	*80.0(22)*	80	80	*10.8(7)*	10.76	10.76
O2	80.0(20)	80	80	*65.1(16)*	65.15	65.15	*60.4(12)*	60.37	60.37	*18.4(12)*	18.38	18.38

Table S2. A list of the refined structural parameters of NVP.

Name	X	Y	Z	U_{iso}	Site	Wyck.	Frac.
**V**	1/3	2/3	-0.180(2)	0.02(2)	3	12c	1
**Na(1)**	1/3	2/3	1/6	0.18(17)	-3	6b	0.8
**Na(2)**	2/3	0.9777(17)	1/12	0.053(5)	2(010)	18e	0.733
**P**	-0.0427(7)	1/3	1/12	0.0170(22)	2(100)	18e	1
**O(1)**	0.1399(5)	0.4964(5)	0.07676(17)	0.0160(15)	1	36f	1
**O(2)**	0.5408(7)	0.8433(7)	-0.0268(18)	0.0362(16)	1	36f	1

Table S3. A list of the refined structural parameters of NVP-$F_{0.15}$.

Name	X	Y	Z	U_{iso}	Site	Wyck.	Frac.
**V**	1/3	2/3	-0.1872(34)	0.065(23)	3	12c	1
**Na(1)**	1/3	2/3	1/6	0.091(12)	-3	6b	0.65
**Na(2)**	2/3	0.9755(16)	1/12	0.047(4)	2(010)	18e	0.733
**P**	-0.0450(7)	1/3	1/12	0.012(2)	2(100)	18e	1
**O(1)**	0.1395(5)	0.4962(5)	0.07647(17)	0.0152(15)	1	36f	0.95
**F(1)**	0.1395(5)	0.4962(5)	0.07647(17)	0.0152(15)	1	36f	0.05
**O(2)**	0.5407(6)	0.8441(6)	-0.02669(16)	0.0320(15)	1	36f	0.95
**F(2)**	0.5407(6)	0.8441(6)	-0.02669(16)	0.0320(15)	1	36f	0.05
